# Development of an in-situ forming implant system for levodopa and carbidopa for the treatment of parkinson’s disease

**DOI:** 10.1007/s13346-025-01892-y

**Published:** 2025-06-07

**Authors:** Deepa D. Nakmode, Sadikalmahdi Abdella, Yunmei Song, Sanjay Garg

**Affiliations:** Centre for Pharmaceutical Innovation, University of South Australia, North Terrace, Adelaide, SA 5000 Australia

**Keywords:** Levodopa, Carbidopa, In-situ forming gel, Long-acting, Sustained release, In-vivo prediction, Biodegradation

## Abstract

Long-acting injectables have gained attraction as a system for treating chronic conditions due to their increased efficacy, safety, and patient compliance. Currently, patients with Parkinsons need to administer oral medications multiple times a day which imposes the significant risk of non-compliance. This study aimed to design an in-situ implant-forming system for controlled delivery of levodopa and carbidopa for up to 1 week which will reduce the need for multiple dosing. The combination of poly-lactic-co-glycolic acid (PLGA _50:50_) and Eudragit L-100 was used to prepare the implants and the formulation was optimized to achieve a controlled release over 7 days. The optimized formulation containing 26% PLGA and 6% Eudragit L 100 displayed a favorable release profile and injectability with low viscosity. The optimized formulation in vitro release study revealed an initial burst of 34.17% and 37.16% for levodopa and carbidopa in the first 24 h and about 92% and 81% release within 7 days. A good correlation was observed between the in-vitro drug release data and ex-vivo drug release with a correlation coefficient of 0.91 for levodopa and 0.90 for carbidopa. Viscosity analysis showed the Newtonian behavior of the formulation. Syringeability analysis of the formulation showed that the maximum force required for expelling the formulation was 32.98 ± 0.72 N using a 22 G needle. The in-vitro degradation studies revealed 81.89% weight loss of implant in 7 days. The prepared formulation was assessed for in-vivo performance using a convolution modeling technique using a convolve function in R software. The predicted AUC 0-∞ h for the in-situ forming implant was 26505.5 ng/ml with Cmax, 399.3 ng/ml, and Tmax 24 h assuming 100% bioavailability. The results justify that the prepared in-situ implant forming system can be a promising system for the delivery of levodopa and carbidopa for Parkinson’s patients.

**Supplementary Information:**

The online version contains supplementary material available at 10.1007/s13346-025-01892-y.

## Graphical Abstract

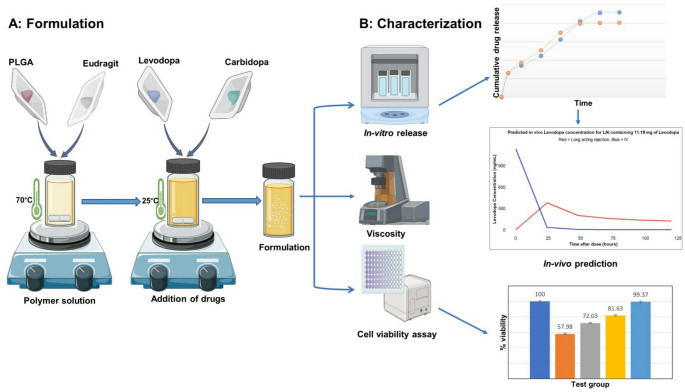

## Introduction

Parkinson’s disease is a progressive neurodegenerative disorder, that causes the loss of dopamine-producing neurons (1). It is the second most commonly occurring neurological disorder and affects 8.5 million worldwide [1]. Currently, there is no cure for Parkinson’s disease however symptomatic treatments consisting of dopaminergic drugs such as levodopa and dopamine agonists are available [2]. Levodopa (LD) was the first approved, most efficient symptomatic treatment for Parkinson’s disease. It has always been given in fixed-dose combination with decarboxylase inhibitor (carbidopa) to prevent its peripheral degradation. Carbidopa (CD) inhibits the peripheral metabolism of levodopa by inhibiting dopamine decarboxylase enzyme [3]. Levodopa is recommended at all stages of Parkinson’s disease as early monotherapy, in patients with or without dyskinesia [4]. Levodopa crosses the blood-brain barrier easily after which it gets converted to dopamine in the brain, Due to the short half-life of levodopa, it requires multiple time administrations [5], which causes fluctuation in the plasma concentration of the levodopa. Previous studies have demonstrated that sustained-release tablets had fewer plasma drug concentration fluctuations compared to immediate-release formulations, highlighting the benefits of controlled-release formulations for the effective treatment of Parkinson’s disease [5]. The existing treatment options including oral formulations require multiple administrations in a day which is difficult for most patients particularly the elderly and people with swallowing difficulty making the treatment of Parkinson’s very challenging. Thus, there is a need for long-acting injectables to maintain constant plasma levels with reduced side effects [6].

Alternative to the existing treatment, long-acting injectables provide sustained release of the drug with constant plasma levels of the drug while reducing the dosing frequency and side effects of the drug [6]. Long-acting injectables include various approaches such as microparticles, liposomes, microneedles, oily solutions, suspensions, oloegels, and solid implants. In addition to this system, an alternative technique is employed for overcoming the issues with microsphere and solid implants such as limited drug loading, inadequate dispersion of microsphere affecting the dosing and syringeability, and requiring surgery for removing the implant [7]. In-situ systems offer easy painless administration using small needles and an easy manufacturing process. In-situ forming implants were first introduced by Dun et al. In 1990, where they developed a biodegradable in-situ forming implant [8]. Classification of in-situ implant system is done based on the mechanism of implant formation, namely in-situ solidifying organogels, in-situ cross-linking system, and in-situ precipitating [7, 9]. In-situ precipitation implants are formed by phase separation due to changes in temperature, pH, or solvent exchange. Solvent-induced phase inversion implants have attracted special attention over other systems as it does not require any change of temperature, or presence of ions for implant formation [10]. These systems are widely used in commercial products for clinical indications. In Table 1 we have summarised the preclinical studies on in-situ forming implants.

**Table 1:** Overview of reported in-situ forming implant formulations along with composition

Drug	Polymer	Molecular weight (Da)	Solvent	Release up to (days)	Indication	References
Meloxicam	PLGA _50:50_	-	N-methyl pyrrolidone	23 days	Rheumatoid arthritis	[11]
Paclitaxel	PLGA _50:50_	7000–17,000 24,000–38,000 38,000–45,000	N-methyl pyrrolidone	28 days	Cancer	[12]
Leuprolide	PLGA _50:50_	25,000	N-methyl pyrrolidone	30 days	Cancer	[13]
Doxycycline hydrochloride Secnidazole	PLA PLGA _50:50_	75,000-120,000 40,000–75,000	N-methyl pyrrolidone	70 h	Periodontitis	[14]
Haloperidol	PLGA _50:50_	60,000–70,000	N-methyl pyrrolidone	28 days	Antipsychotic	[15]
Enfuvirtide	PLGA _50:50_	6,000	Dimethylsulfoxide	48 h	HIV infection	[16]
Dexamethasone	PLGA _50:50_	15,000	N-methyl pyrrolidone	35 days	Diabetic retinopathy, Age-related macular degeneration	[17]

The in-situ implant forming systems also provide high drug loading capacity with a specific release rate due to the low surface area available for drug release in comparison to the microsphere systems. In contrast to delivery systems for subcutaneous or intramuscular injection, the fewer restrictions on the size requirement for an implant could provide an additional advantage by allowing the delivery of larger doses of drugs [18].

The composition of solvent-induced phase separation systems includes the water-insoluble polymer, which is dissolved in an organic biocompatible solvent (water miscible) and drug either in dispersed or dissolved form [13]. Upon administration of the formulation, the solvent dissipates into the aqueous environment, followed by diffusion of water leading to precipitation of polymer resulting in implant formation [19]. Some of the commercial formulations that work on the mechanism of in-situ forming implants include Atridox 450 mg, Atrigel and Eligard 30 mg [20].

In the current study, we have developed an in-situ forming implant for levodopa and carbidopa for the treatment of Parkinson’s disease. The current therapies available for treating Parkinson’s provide symptomatic treatment only. Amongst these levodopa remains most frequently prescribed for treating bradykinesia, and rigidity [21]. To avoid peripheral side effects of levodopa such as nausea, it is usually given with carbidopa or benserazide [22]. Currently, levodopa is not available in the parenteral sustained release form, hence there is a need for a formulation that will provide sustained delivery of levodopa for a longer period, thereby reducing the dosing frequency and improving compliance [6]. In-situ forming implants provide sustained delivery of drugs with the added advantage of easy administration due to their low viscosity.

In this study, we have used Poly (lactide-co-glycolide) (PLGA), an FDA-approved biodegradable polymer. PLGA dominates the list of materials used to create microspheres and in-situ implants, and about 46% of marketed long-acting formulations are based on PLGA [23]. The potential advantage of PLGA is that drug release can be modified by using different molecular weights PLGA, altering the drug-to-polymer ratio, size of the microsphere, ratio of glycolic acid to lactic acid ratio, and terminal group of the polymer [24]. Due to their excellent biocompatibility [25], formulation flexibility, different degradation rates [26], and successful clinical record, PLGA copolymers have been the primary ingredient in most LAI technologies that have been introduced in recent years. PLGA is a hydrophobic polymer, used to modify the release of drugs from PLGA in-situ forming gels. The addition of a hydrophilic polymer helps in achieving the complete release of the drug from the implant. In this study, we have used PLGA _50:50_ acid terminated of lower molecular weight as PLGA 50:50 degrades faster than the other grades of the PLGA [27]. For achieving 1 week release PLGA 50:50 was selected for the formulation.

Another polymer blended with PLGA was Eudragit L-100, as combining polymers with diverse physicochemical properties such as solubility, viscosity, and glass transition temperature can help in altering the release profile of the drug. The class of Eudragit polymers is a good candidate for blending with hydrophobic polymers because of the extensive range of grades with interesting applications in the formulation [28]. Particularly, Eudragit L-100 which dissolves above pH 6, is advantageous for intramuscular (IM) formulation. In-situ forming implants using PLGA and Eudragit L-100 have not been reported in the literature. Different ratios of PLGA and Eudragit L-100 were optimized for achieving the targeted release profile. P5 formulation gave an optimum release profile. The optimized formulation was evaluated for viscosity, syringe ability, and in-vitro and ex-vivo drug release. To prove the biocompatibility of the prepared formulation, cell viability (MTT assay) was also performed.

## Materials and methods

### Materials

PLGA _50;50_ polymers (M.W– 15,000 Da, acid end cap) were purchased from Nomisma Healthcare (Gujrat, India). Polyethylene glycol 400 (PEG-400) was purchased from Sigma Aldrich. N, N-dimethyl acetamide was purchased from Sigma Aldrich (MO, USA), Sodium bisulfite was obtained from Sigma Aldrich (Capital, India), Eudragit L-100 was purchased from Evonik (Darmstadt, Germany), Tween 80 was purchased from Chemsupply (Gillman, Australia), Acetonitrile was purchased from Sigma Aldrich (Darmstadt, Germany), Trizma base was purchased from Sigma life science (MO, USA) and Trizma HCL was purchased from Sigma Aldrich (MO, USA). N-methyl pyrrolidone was purchased from Chemsupply (Gillman, Australia).

### Preparation of in-situ implant formulations

**Fig. 1:**
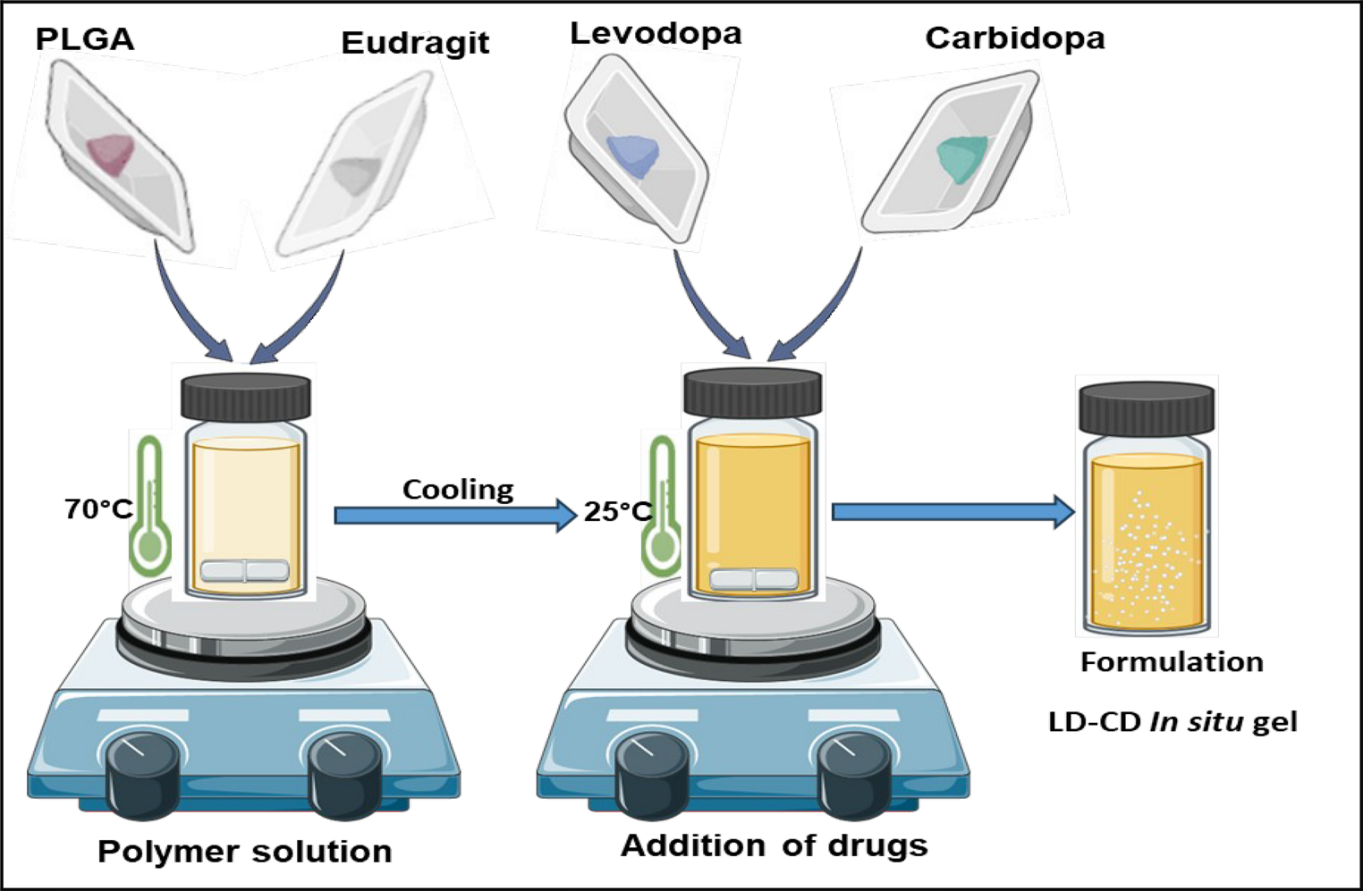
Preparation of in-situ forming implant formulation

Polymeric solutions were prepared by dissolving PLGA (P1 to P4) and Eudragit L 100 (P5) in organic solvent N, N-Dimethylacetamide (DMAC) or N-methyl pyrrolidone (NMP) at 70 °C, with constant stirring at 700 RPM. Once the clear solution was formed, a measured volume of PEG 400 was added until a homogenous solution was obtained. Once the homogenous solution was formed, heating was stopped weighed an amount of levodopa and carbidopa (4:1 ratio) were added to these solutions under continuous stirring for 30 min (illustrated in Fig. 1). All formulations contain 8.75% of levodopa and 2.18% of carbidopa suspended in the polymeric solutions. The prepared final formulation was a suspension. The prepared formulations were stored at 5 ± 3 °C until the characterization was performed. Different ratios of PLGA to Eudragit L 100 were tested for their effect on the drug release profiles of levodopa and carbidopa. The formulation trials carried out with different ratios of PLGA are listed in the Table 2 which were further tested for in-vitro drug release. The optimized formulation P5 was further evaluated for FTIR, Viscosity, Syringeability, ex-vivo release, in-vitro degradation, biocompatibility, and in-vivo performance prediction was performed.

**Table 2:** Formulation trials for in-situ forming implant

Composition (grams)	P1	P2	P3	P4	P5
PLGA	1.2	1.2	1	1	1.04
Eudragit L100	0	0	0	0	0.24
Sodium bisulfite	0.008	0.008	0.008	0.008	0.008
Dimethylacetamide	1.33	0	1.33	0	1.33
N-methyl pyrrolidone	0	1.33	0	1.33	0
PEG-400	1.458	1.458	1.658	1.658	1.38
Levodopa	0.350	0.350	0.350	0.350	0.350
Carbidopa	0.0875	0.0875	0.0875	0.0875	0.0875

### Characterization of formulations

#### Drug content (assay) determination

The drug content of all the formulations was determined by weighing 24 mg of each formulation into the volumetric flask and adding 500 µl DMAC to dissolve PLGA completely. The mixture was vortexed, and the volume was adjusted to 20 ml with 0.1 N phosphoric acid. All the samples were kept on bath sonication for 1 h to allow extraction of both the drugs. All the samples were filtered through a 0.45 μm PVDF filter and then analyzed by HPLC at 280 nm wavelength. The results were mentioned at mean ± SD (*n* = 3).

#### In-vitro drug release testing

In-vitro drug release was performed by adding ~ 100 mg of each formulation into 10 ml of tris buffer pH 7.4 with 0.2% v/v tween 80 in a clear glass vial [29]. The in-situ implant was formed instantaneously after the addition of formulation into the buffer. All the vials were kept in an incubator at 37 °C at 250 RPM, at each time point the release media was replaced completely with fresh buffer to maintain the stability of the drugs. The collected release samples were stored at -20° C until analysis by HPLC [30].

#### Mathematical models

Based on the in-vitro drug release information, the release kinetic of levodopa and carbidopa from the formulation was assessed. The release data fitting was performed for various release models via the Microsoft Excel add-in software program DDsolver. The best-fitted model is selected based on the adjusted coefficient of determination (R^2^_adjusted_) and Akaike information criterion (AIC) [31]. While comparing different models with various parameters R^2^_adjusted_ is considered more meaningful. The model with the highest value for R^2^_adjusted_ and smaller AIC values is illustrated as the best-fitting model [31].

#### pH measurement of the release media

The pH of the release media containing in-vitro drug release formulation was measured to observe the change in the pH of the media, which could contribute to the release of the drug. The pH of the release samples was measured every day for up to 1 week, the studies were performed in triplicates. The release media was replaced every day with a fresh buffer for maintaining the stability of the drugs.

#### Fourier transform infrared spectroscopy (FTIR)

For confirming the compatibility of the drugs with polymeric solution FTIR spectra were recorded using an FTIR spectrometer (Bruker, Massachusetts, USA). For recording the FTIR spectra each sample was placed on the ATR diamond crystal in a small amount with the application of a clamp for ensuring proper contact between the sample and crystal. All the recordings were performed at room temperature in transmittance mode in the range of 4000 to 450 cm ^-1^ with 16 scans per sample.

#### Viscosity measurement

For evaluating the rheological behaviour of the formulations Rheosys Merlin VR [32] (Scientex Pty Ltd, Melbourne, Victoria, Australia) was used with a parallel plate of diameter 30 mm with a 0.5 gap [33]. The observed viscosity was plotted against the shear rate (1/s).

#### Syringeability and implant formation evaluation

**Fig. 2:**
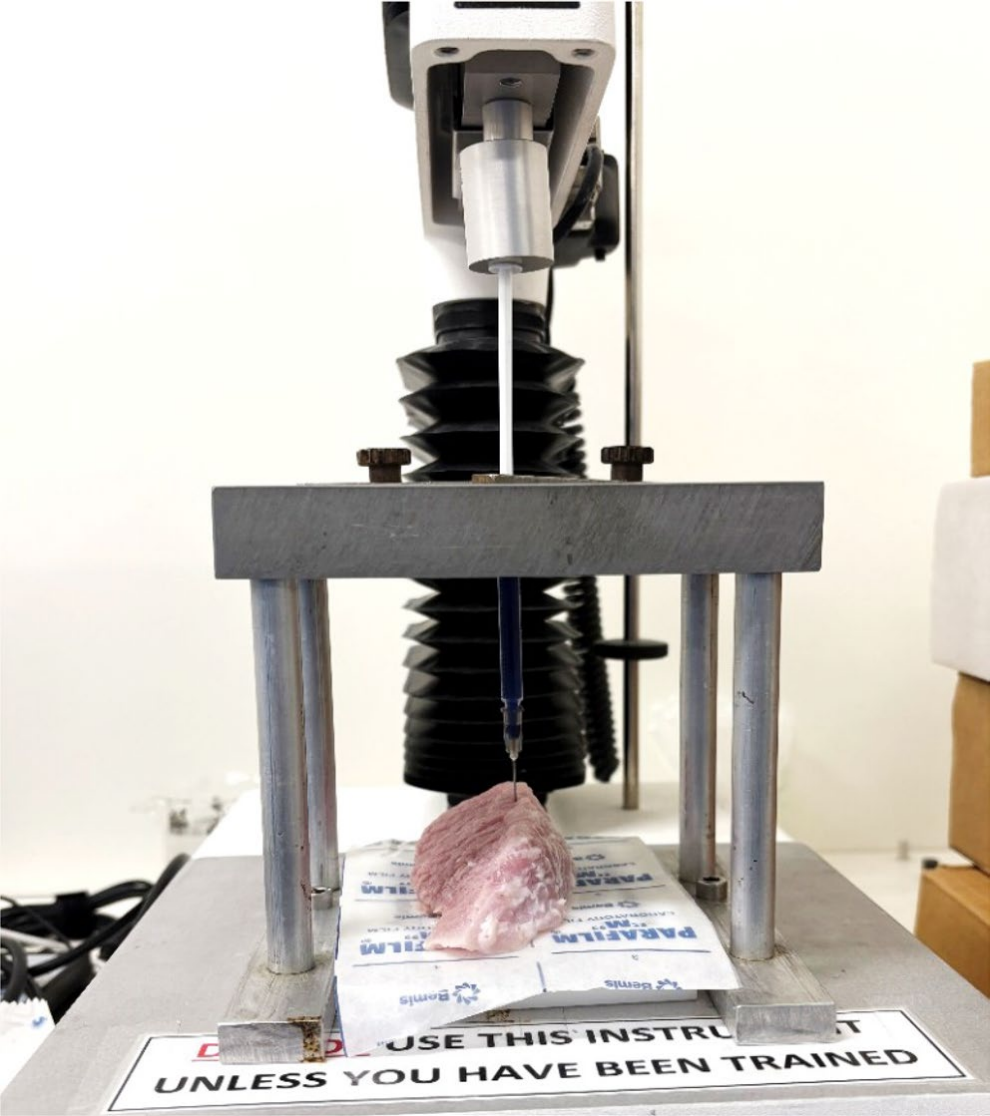
Syringeability testing using a texture analyzer

Syringrability in the muscle was performed using the pig hind leg muscle of thickness 4 cm. The texture analyzer (TA. XT plus texture) was used in compression mode where the prepared formulation was drawn into a 1 ml syringe fitted with a 22 G needle. The pig’s hind leg muscle was placed at a 90 ° angle to the syringe as shown in Fig. 2. The parameters such as distance and a test speed of compression were kept at 30 mm and 1.00 mm/sec respectively [34] and evaluation was performed in triplicates at room temperature. The force required to expel the formulation is expressed in newtons (N). For confirmation of depot formation, trypan blue dye was added to the formulation. The muscle was cut open after five minutes of injecting the formulation and observed visually for implant formation.

#### Scanning electron microscopy (SEM)

The structural morphology of implants formed in-vitro was studied using scanning electron microscopy. Implants were formed in-vitro by injecting 100 µl of formulation into 10 ml of pH 7.4 buffer [32]. On different time intervals (Day 1, 4, and 7) implants were taken out of the buffer and frozen with liquid nitrogen followed by freeze-drying (Labanco freeze dryer) for 72 h. The cross-section of freeze-dried implants as well as the implant as such were observed under the microscope [35]. All the samples were placed on aluminium stubs followed by coating with platinum using Agar high-resolution sputter. SEM micrographs were obtained for all the samples using Zeiss Merlin FEG SE.

#### Degradation studies

The in-vitro degradation studies were performed to estimate degradation time for in-situ formed implants. The degradation of in-situ formed implants was performed by adding 100 mg of formulation in pH 7.4 buffer which was incubated at 37 °C with stirring at 250 rpm. The implants were removed from the buffer at regular intervals and the extra water was eliminated with the help of tissue paper followed by weighing the implant. The percentage weight loss was calculated by using the following formula [36]:

$$\:Degree\:of\:degradation\:\left(\%\right)=\frac{Wt}{Wi}\times100$$

Where W_t_ is the weight of the degrading implant and W_i_ is the initial weight of the implant.

#### Cell viability study (MTT assay)

Sterilized in-situ gel was evaluated for cytotoxicity concerning the viability, and growth of fibroblasts using 3T3 fibroblast cells and an MTT assay [37]. 3T3 cells are the standard fibroblastic cells that are commonly present in the various connective tissues and are generally used for testing the biocompatibility of the biomaterials [37]. MTT assay is often a gold standard for testing the cytotoxicity compared to another available assay [38]. Cytotoxicity studies were carried out using 3-(4,5-dimethylthiazol-2-yl)-2,5-diphenyltetrazolium bromide MTT assay [39, 40]. Cytotoxicity assay was conducted according to ISO 10993-5 standards. Fibroblasts of line NIH 3T3 were grown in DMEM media supplemented with 10% FBS, and 1% penicillin streptomycin with 5% CO_2_ at 37 °C. For cell viability study 100 mg of the in-situ implant was extracted at 37 °C for 12 h in a 10 ml DMEM media. The extracted samples were further diluted as 2X,5X,10X, and 20X using DMEM media for treating cells. 100 µL of 3T3 cells were seeded into the 96 well plates at a density of 5,000 cells per well and incubated for 24 h. Extracts were placed on the mouse fibroblast cell line NIH 3T3 which were examined for cytolysis. After treating cells with extract and incubation at 37 °C for 24 h. Serum-free medium of 90 µl and 10 µl MTT (5 mg/ml) was added to each well followed by incubation for 3 h at 37 °C until the formation of formazan crystals occurred. Then 150 µL of Dimethyl sulfoxide (DMSO) was added to each well for dissolving the formazan crystal and absorbance was measured at 540 nm using a PerkinElmer Wallac plate reader (PerkinElmer, Inc., Waltham, MA, USA) [41]. The cell viability was calculated using the following formula:

$$\:Cell\:viability\:\left(\%\right)\:=\:\frac{OD\:sample}{OD\:control}\times100$$

OD- optical density.

#### Ex-vivo release studies and In-vitro Ex-vivo correlation

The ex-vivo release studies were performed in triplicates using the pig hind leg muscle of size ∼ 7 cm by 5 cm size. The formulation was filled into the 1 ml syringe with a 22 G needle injected into each muscle (300 µL formulation) and the muscle was transferred to the beaker containing 60 ml of tris buffer pH 7.4 with 0.1% sodium azide. All the beakers were kept at 37 °C with agitation of 250 RPM. The release samples were collected at each time point and release media was completely changed every 24 h. The release samples were analyzed by HPLC [30]. The in-vitro drug and ex-vivo were fitted using linear regression in Excel.

#### Prediction of in-vivo performance

The in-vivo performance of the optimized formulation was predicted by the convolution approach. Based on the in-vitro drug release data and the pharmacokinetics of the intravenous levodopa injection obtained from the literature [42] the in-vivo pharmacokinetics were predicted. The convolution was performed using the convolve function in the R programming language [43]. Convolution is an integration process that involves modeling the in-vivo plasma drug concentrations obtained on the administration of a unit dose of a formulation. Convolution-based modeling involves the identification of Unit input Response (UIR) data for the drug which characterizes the pharmacokinetics of the drug. Convolution is an integration process that involves modeling the in-vivo plasma drug concentrations obtained on the administration of each formulation dose unit. This is further combined with the fraction dissolved in-vitro for each dosage unit in a single step that integrates the convolution integral.

$$\:C\:\left(t\right)=\:{\int\:}_{0}^{t}I\:\left(x\right).u\left(t-x\right)dx$$

Where C (t) is the plasma concentration of the drug at time t following administration of the formulation. If I(t) is the rate at which the drug is released from the formulation at time t and the UIR is denoted by U (t) which is the plasma concentration-time relationship, then the plasma concentration of the drug C (t) from the entire dose I(t) is given by above equation [43].

## Results and discussion

### Preparation of the in-situ forming implants

**Fig. 3:**
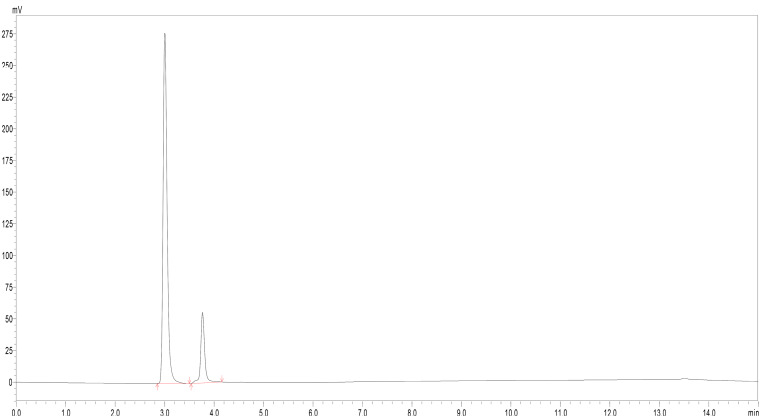
Chromatogram demonstrating separation (**a**) Levodopa (Rt: 3.06 min) and (**b**) Carbidopa (Rt: 3.74 min) from the formulation

Firstly, PLGA gel was prepared in DMAC and NMP and evaluated for drug release, the gels containing DMAC as solvent showed consistent drug release whereas NMP-containing gels showed very high initial burst release followed by lag phase. When the concentration of PLGA was reduced indistinguishable difference was observed (Fig. 3). Due to the hydrophobic nature of PLGA, very slow drug release was observed in reducing the concentration of PLGA, and high initial burst release was observed. Therefore, to modulate the drug release with reduced initial burst release, the combination of PLGA and other hydrophilic polymers was selected and evaluated for effect on the drug release profile. The gels were prepared using a 9:1 ratio of PLGA with Pluronic F-68, Pluronic F-127, Pluronic F-108, HPMC K4, Eudragit L-100, Eudragit E 100, and PEG 4000.

Drug release studies were carried out on all the developed formulations. Except for Eudragit L-100, all the formulations showed burst release with more than 60% drug release in 24 h, however with Eudragit L-100 lower burst release with consistent drug release was observed. Hence Eudragit L100 was selected for preparing gels and evaluated for its effect on drug release at different concentrations in combination with PLGA. For dissolving polymers DMAC and PEG-400 were used as using DMAC alone in higher concentrations could be harmful for the IM route. Also, the addition of PEG-400 helps in maintaining the viscosity. DMAC and PEG-400 are miscible with each other [44]. The optimized formulation was obtained at 26% PLGA and 6% Eudragit L-100. The composition of the optimized formulation is given in Table 1. The optimized formulation was evaluated under storage conditions.

### Drug content determination

The drug content for levodopa was found to be 2.02 ± 0.21 mg, whereas for carbidopa it was found to be 0.59 ± 0.04 mg in a 24 mg formulation. Figure 3 represents the chromatogram of levodopa and carbidopa after extraction from the formulation analyzed by HPLC. The extraction method was validated by using the pure drug as well as the blank gel.

### In-vitro drug release studies

**Fig. 4:**
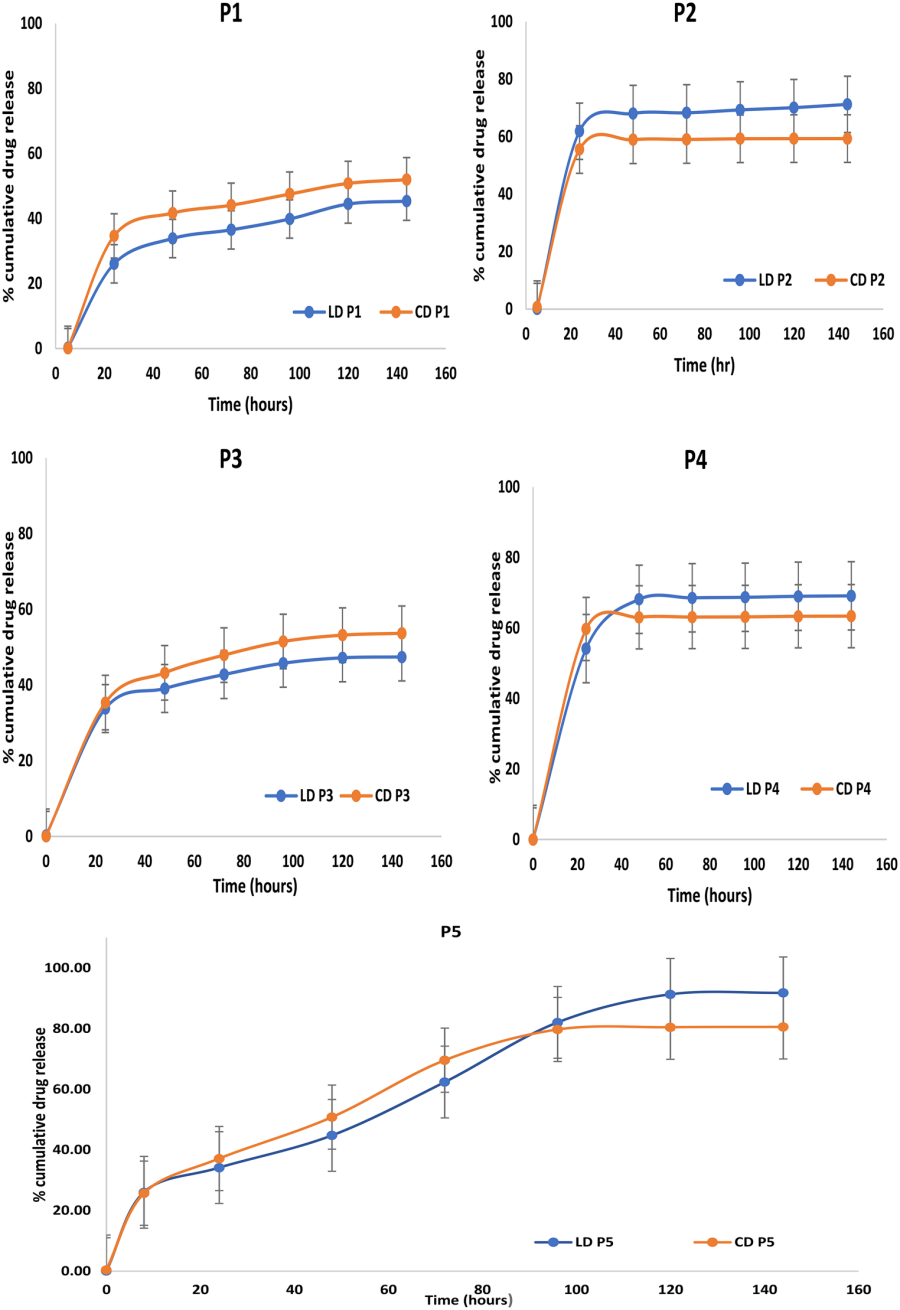
In-vitro drug release data for levodopa and carbidopa in-situ implant formulation (Mean ± SEM, *n* = 3)

The in-vitro drug release data from gels containing DMAC (P1 and P3) at different concentrations of PLGA showed less burst release of (26.04% and 33.78% levodopa) in 24 h followed by the consistent release of drug over 1 week as shown in Fig. 4. On the other hand, P2 and P4 containing NMP as solvent showed very high burst release (61.88% and 54.15%) followed by steady release up to 59.33 and 69.12% of levodopa in 144 h. Therefore, a formulation containing DMAC was selected due to consistent release with less initial burst release, which was further modified with the addition of a hydrophilic polymer to optimize the drug release.

The optimized formulation (P5) showed 20.36% levodopa release in the first 8 h followed by 34.17% release of levodopa in 24 h, whereas 25.72% carbidopa was released in the first 8 h followed by 37.16% drug release in 24 h. Slow and continuous release was observed for up to 7 days. Release studies were continued for 7 days, 92% cumulative levodopa was released in 144 h, whereas up to 80% carbidopa was released in 144 h. As the drug was in the suspended form in the polymer solution, the release mechanism could be attributed to the diffusion of the drug from the formulation into the release media.

### Mathematical modeling

Prediction of drug release kinetics has been widely done using mathematical models. The coefficient of determination (R^2^) and AIC (Akaike information criterion) can be used for model fitting and determining a suitable model for the formulation. The amount of drug released from the formulation plays an important role in maintaining the therapeutic concentration of the drug. The best-fitting model is the one with the highest value of R^2^ and lowest AIC [45]. The release kinetics of levodopa and carbidopa were modeled with zero order, first order, Higuchi model, Kormeyer-Peppas equation, Makoid-Banakar model, and Peppas Sahlin model as shown in Table 3. The models with R^2^ ≥ 0.97 and AIC near ≤ 50 were considered adequate.

**Fig. 5:**
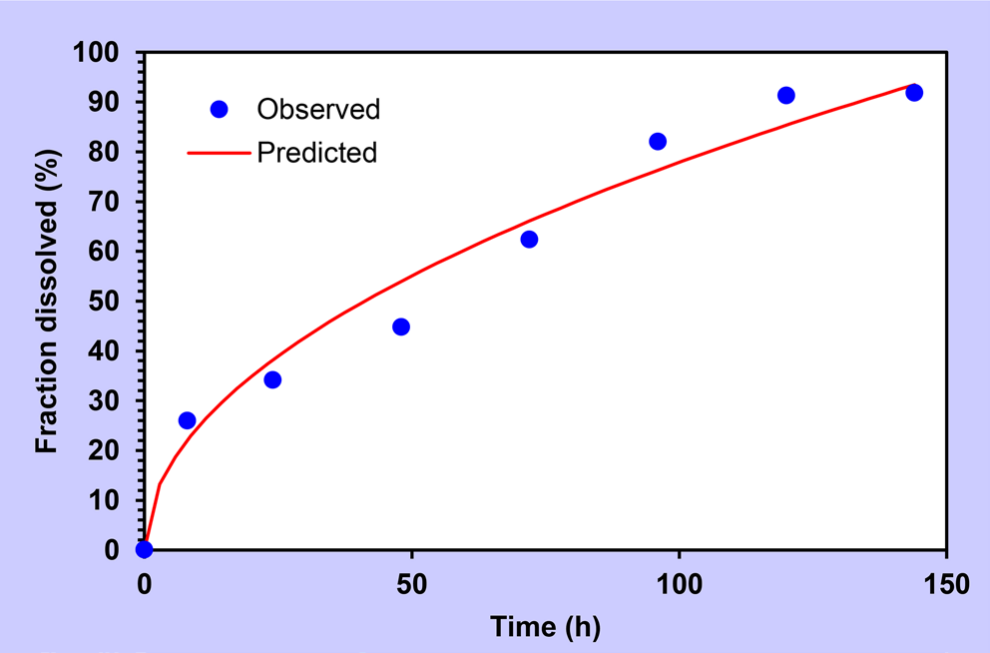
A release parameter fitted for levodopa for in-situ gel by Higuchi model

**Fig. 6:**
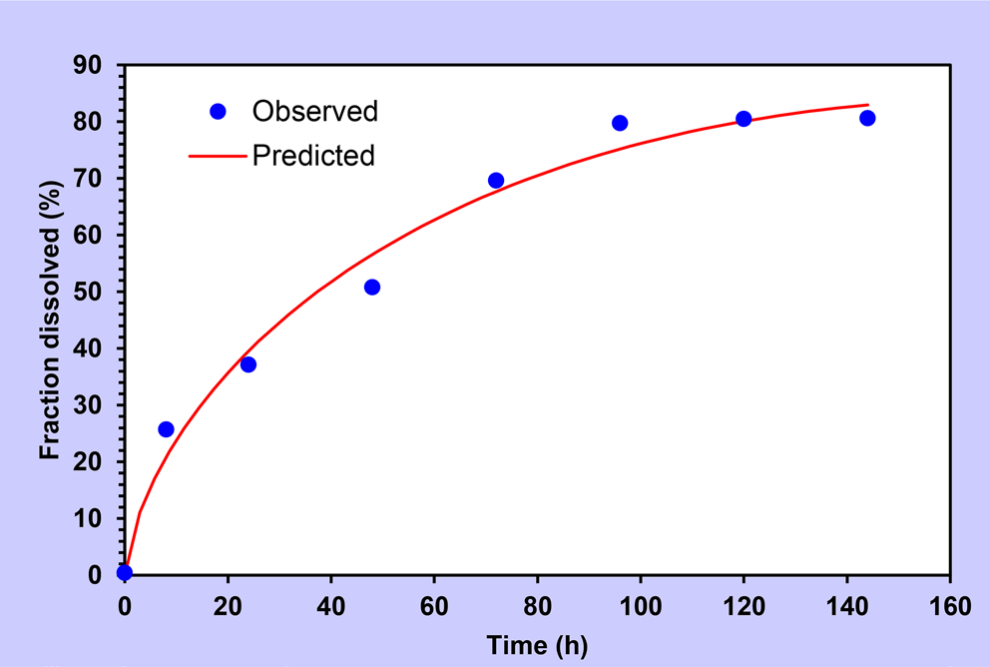
A release parameter fitted for Carbidopa for in-situ gel by Peppas Sahlin model

The best-fitting model for levodopa was the Higuchi model as shown in Fig. 5 [31, 46], whereas for carbidopa Peppas-Sahlin model showed the best fitting as represented in Fig. 6.

The Higuchi model is represented by the following equation [47]:

$$F\,=\,{K_2}{t^{1/2}}$$

Where F is the fraction of the drug released, K_2_ is the Higuchi dissolution constant, and t is the time at drug is released. A number of models were developed by Higuchi explaining the release mechanism of water-soluble and low-water-soluble drugs, which were dispersed in the uniform matrix that behaves as the diffusion media [46]. The Higuchi model defines the mechanism of drug release as diffusion based on Fick’s law which states the rate of drug release is proportional to the square root of time [31]. This model is widely applied for describing the drug release mechanism from the modified release products [48]. Based on the mode fitting levodopa seems to obey the Higuchi diffusion model responsible for drug release from the implant matrix [49].

Peppas Sahlin model is represented by the following equation:

$$F\,=\,{K_1}{t^m}\,+\,{K_2}{t^{2m}}$$

Where K_1_ is the Fickian kinetic constant, K_2_ erosion rate constant, m is the diffusional exponent and t is the time of drug release. if K_1_/k_2_ > 1, then release occurs due to the Fickian diffusion, if K_1_/k_2_ < 1 by erosion, and if K_1_/k_2_ = 1, then both mechanisms are responsible for the release [9]. For Carbidopa, K_1_/k_2_ was observed to be < 1 which indicates that the release of carbidopa occurs due to the erosion of the polymer [50].

**Table 3:** Mathematical modeling for release kinetics of levodopa and carbidopa

Drug	Model	Equation	*R*^2^_adj	AIC	MSC
Levodopa	Zero-order	F = k0 × t	0.850	58.53	1.65
	First order	F = 100 × [1 − Exp (− k1 × t)]	0.953	49.25	2.88
	Higuchi model	F = K2t1/2	**0.974**	**44.45**	**3.41**
	Korsmeyer-Peppas	F = kkP × tn	**0.973**	**45.44**	**3.28**
	Peppas-Sahlin	F = k1 × tm + k2 × t2m	**0.970**	**46.76**	**3.12**
Carbidopa	Zero-order	F = k0 × t	0.67	63.07	0.86
	First order	F = 100 × [1 − Exp (− k1 × t)]	0.946	48.58	2.67
	Higuchi model	F = K2t1/2	**0.970**	**43.87**	**3.25**
	Korsmeyer-Peppas	F = kkP × tn	**0.975**	**43.07**	**3.35**
	Peppas-Sahlin	F = k1 × tm + k2 × t2m	**0.979**	**42.28**	**3.45**

### pH measurement of release media

The change in the pH of the release media containing formulation was measured immediately after adding formulation as well as every day up to day 7. The results are presented in Table 4. On day 2, the pH of the release media dropped to 3.87 which could be due to the degradation of the PLGA molecule. PLGA is made up of several hydroxyl acid monomers such as d-lactic acid, l-lactic acid, and glycolic acid. The hydrophobicity, release rate, and degradation rate of PLGA are determined by the lactic acid content and molecular weight, higher lactic acid content will degrade over a longer period with the slow-release [51].

The hydrolytic cleavage of the ester linkages in the initial stage of PLGA’s degradation results in the formation of oligomers and monomers. During this time, huge amounts of lactic and glycolic acids are formed, which causes pH values to significantly decrease [27, 52]. The decrease in pH of the release media also helps in the release of levodopa and carbidopa from the in-situ formed implant as well as maintenance of stability of the drug in the release media.

**Table 4:** pH changes of the release media over time

Time	Day 1	Day 2	Day 3	Day 4	Day 5	Day 6	Day 7
pH average ± SD	7.32 ± 0.02	3.87 ± 0.09	4.01 ± 0.13	6.1 ± 0.06	6.44 ± 0.35	6.14 ± 0.13	6.48 ± 0.17

### Fourier-transform infrared (FTIR) of formulation

**Fig. 7:**
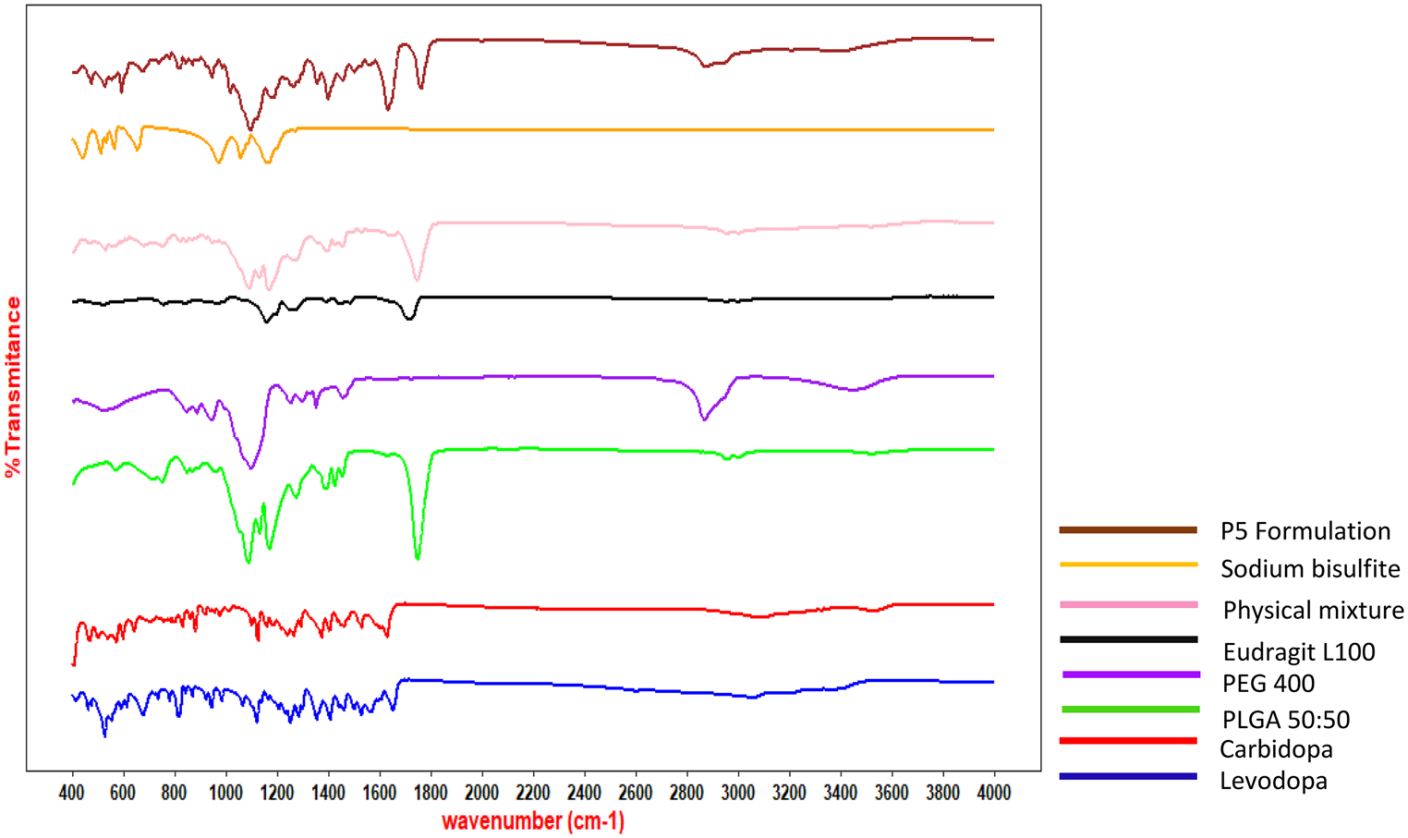
FTIR spectrum of formulation and formulation components

FTIR analysis was carried out to assess the potential interactions of levodopa and carbidopa with the formulation components (Fig. 7). The characteristic peaks were observed for levodopa at 3200, 3062, and 2981 due to O-H stretching and the phenyl group C = C vibrations were observed at 1458, 1402, 1353, 816, and 672 cm^-1^. The C-H stretching was observed at 2929 and 2981 cm^-1^ [53]. At 1562 cm^-1^ NH_2_ stretches were observed and C = O stretching is visible at 1651 cm^-1^. For carbidopa characteristic peaks were observed at 1527 and 1124 cm^-1^ due to NH and NH_2_ stretching and O-H stretching arose at 3518, 3322, and 3289 cm^-1^ [53]. The phenyl group C = C vibrations were observed at 1458, 1402, 1261, 878, and 829 cm^-1^ for carbidopa. The peaks observed at 3102, 3060, and 1373 cm^-1^ are due to C-H stretching. The characteristics peaks for PLGA were O-H stretching at 3512, C-H peak at 2952 cm^-1,^ A strong peak was observed at 1745 cm^-1^ due to C = O stretching, Due to C-O-C stretching strong peak was observed at 1085 cm^-1^ [54].

Eudragit L 100 shows a strong peak at 1712 cm^-1^ due to the vibration of the esterified carboxyl group. Peaks observed at 1155 and 1254 cm^-1^ are from the ester vibrations. The peak observed at 3200 cm^-1^ was due to O-H vibrations and 1450 and 2952 cm^-1^ were due to CHx vibrations [55].

Compared to the spectra of pure levodopa and carbidopa with formulation peaks the O-H peak of PLGA and carbidopa merged with a broad peak at 3403 cm^-1^. The peak at 3202 cm^-1^ was observed due to O-H stretching of levodopa and Eudragit L100. The peak was shifted a little to 3062 and 2941 cm^-1^ due to O-H stretching of levodopa and carbidopa. The phenyl group C = C vibration of levodopa and carbidopa overlapped at 1455 and 1399 cm^-1^. The strong peak appeared at 1758 cm^-1^ in formulation due to the merging of C = O stretching of PLGA and Eudragit L100. The peak of C-O-C stretching of PPLGA was observed at 1092 cm^-1^ in the formulation. The characteristic N-H peak for levodopa was present at 1562 cm^-1^ whereas for carbidopa NH_2_ peak was observed at 1524 cm^-1^ and NH peak at 1122 cm^-1^.

### Viscosity measurement

**Fig. 8:**
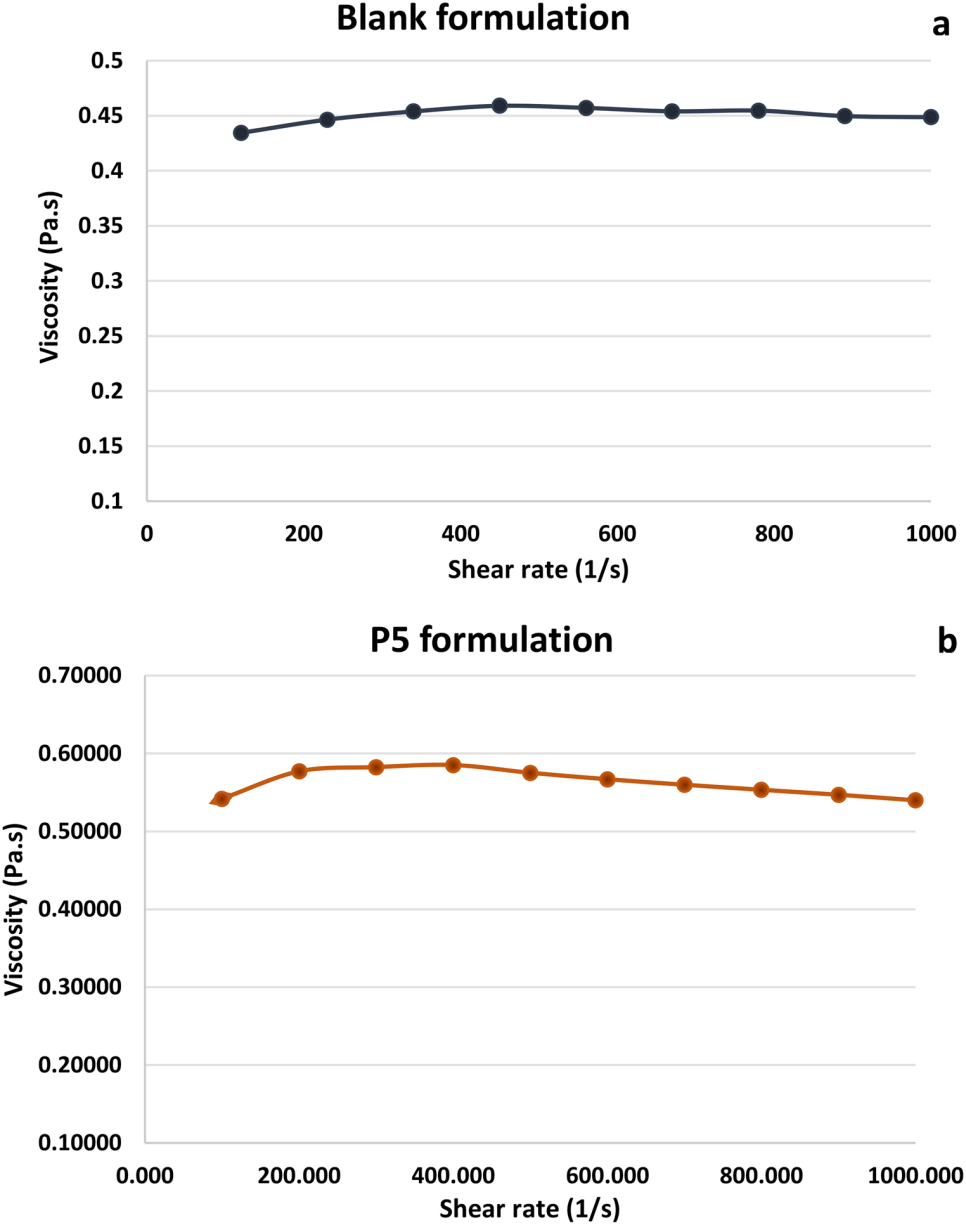
Effect of shear rate on the viscosity (**a**) Blank formulation and (**b**) P5 formulation

Viscosity measurement of the blank formulation (without drug) and formulation was carried out at different shear rates to evaluate the impact of shear rate on the viscosity of the sample. Both the samples showed no major difference in the viscosity when exposed to the shear rate from 100 to 1000 as shown in Fig. 8a below. Where polymer solution, as well as formulation, were freely flowing with low viscosity also called Newtonian behavior [56, 57]. The low viscosity of the formulation indicates good injectability of the formulation [58, 59] which was confirmed by the syringe ability testing. This also demonstrates the selected solvent as a good solvent where polymer-solvent interactions predominate over the polymer-polymer ones when the viscosity is lower [59]. The viscosity of the optimized formulation was also recorded at a different temperature to evaluate the impact of temperature on the viscosity. At room temperature viscosity at the shear rate (1/s) 100 was found to be 0.567 (Pa.s) whereas, at 37 °C, viscosity dropped to 0.338 (Pa.s) which demonstrates that with an increase in temperature, the viscosity of the formulation was found to be reduced.

### Syringeability and ex-vivo implant formation

**Fig. 9:**
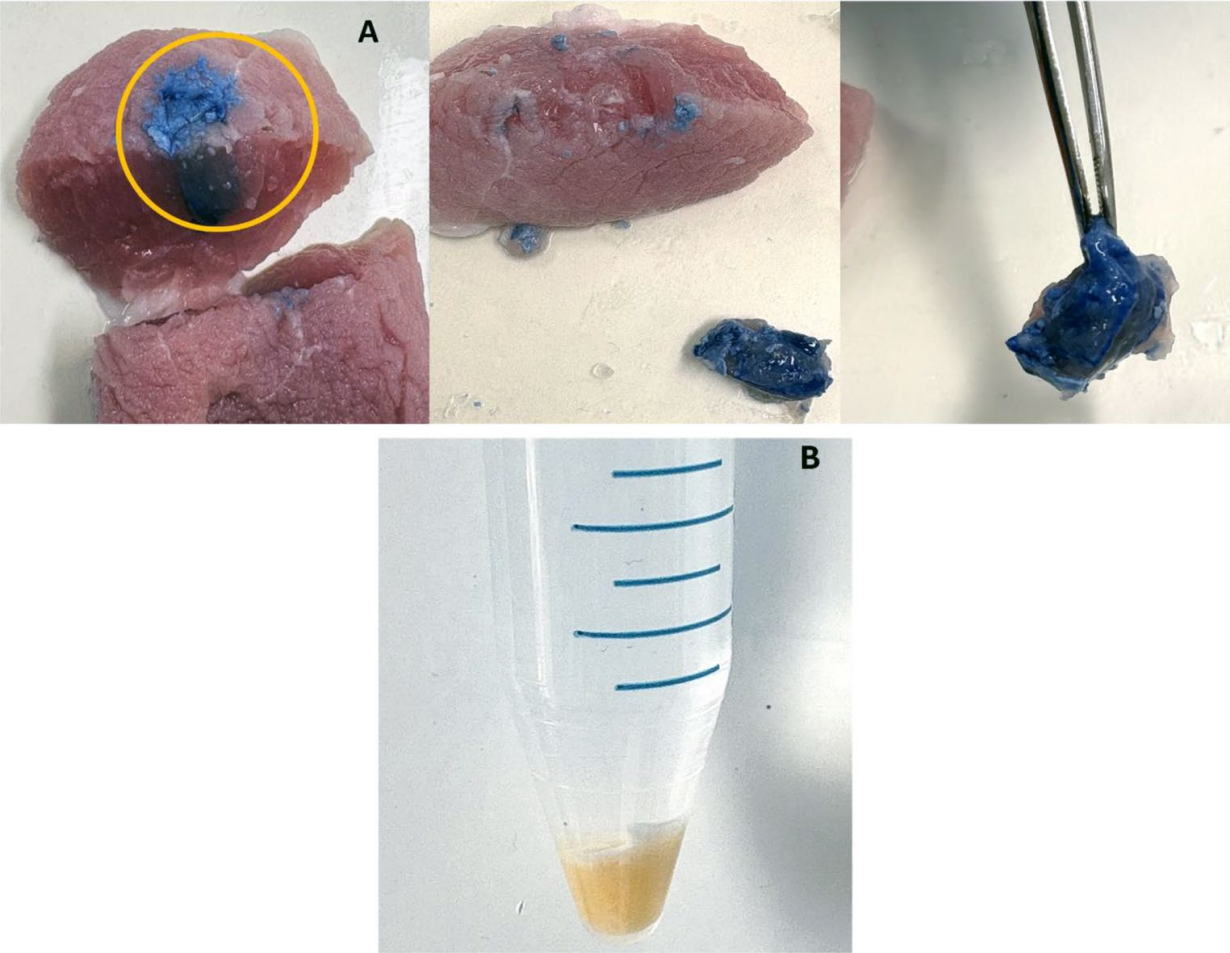
**A** Illustration of implant formation inside pig hind leg muscle and **B** Formation of an implant in tris buffer pH 7.4

One of the important criteria for the injectables is syringeability, the ideal formulations showing smooth ejection are known to possess good injectability (25). Syringeability is the maximum force required for expelling the formulation through the syringe and it’s a crucial parameter for injectable formulations (26, 27). Syringeability of the injectable formulations is the force required to inject or eject the formulation from the syringe, whereas lower the force needed to expel the formulation better the syringeability. The average force required to expel the formulation was found to be 8.02 ± 0.47 N with a 22 G needle, which is within the acceptable maximum injectable force of 40 N [60]. Figure 9 illustrates the implant formation inside the pig’s hind leg muscle. The formation of a solid blue-colored depot was observed, which confirms the formation of a depot inside the muscle.

### Ex-vivo release studies and in-vitro ex-vivo correlation

**Fig. 10:**
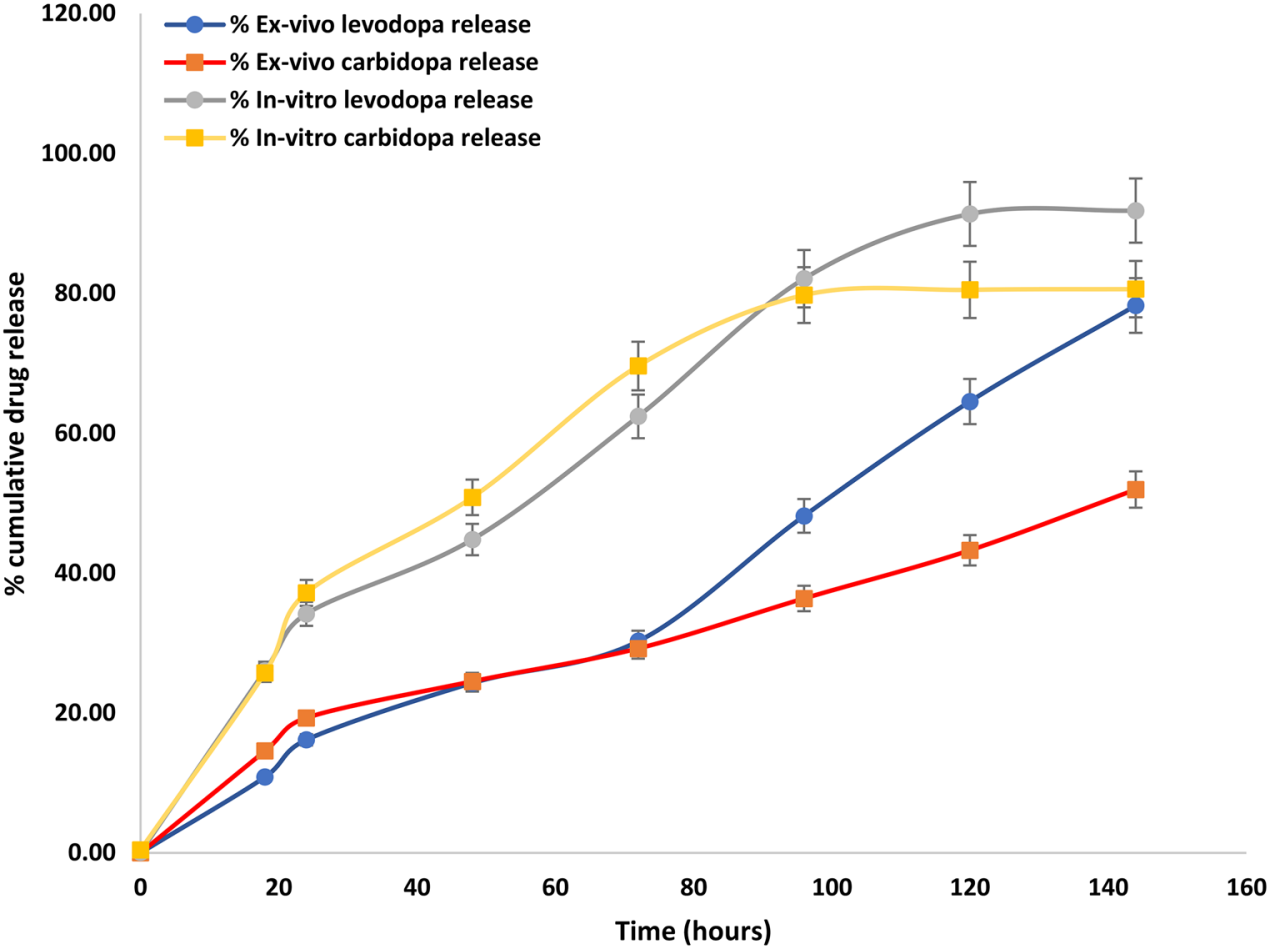
Cumulative ex-vivo and in-vitro drug release profile (Mean ± SEM, *n* = 3)

The ex-vivo release in the case of levodopa was up to 78% drug release observed in 7 days from the muscle whereas with carbidopa up to 51% drug release was observed as shown in Fig. 10. The ex-vivo drug release profile followed a similar release pattern to that of the optimized formulation (P5), However, some differences might be due to differences in the environment between the in-vitro systems and the tissue, implant formed in-vitro might show higher release compared to the muscle.

**Fig. 11:**
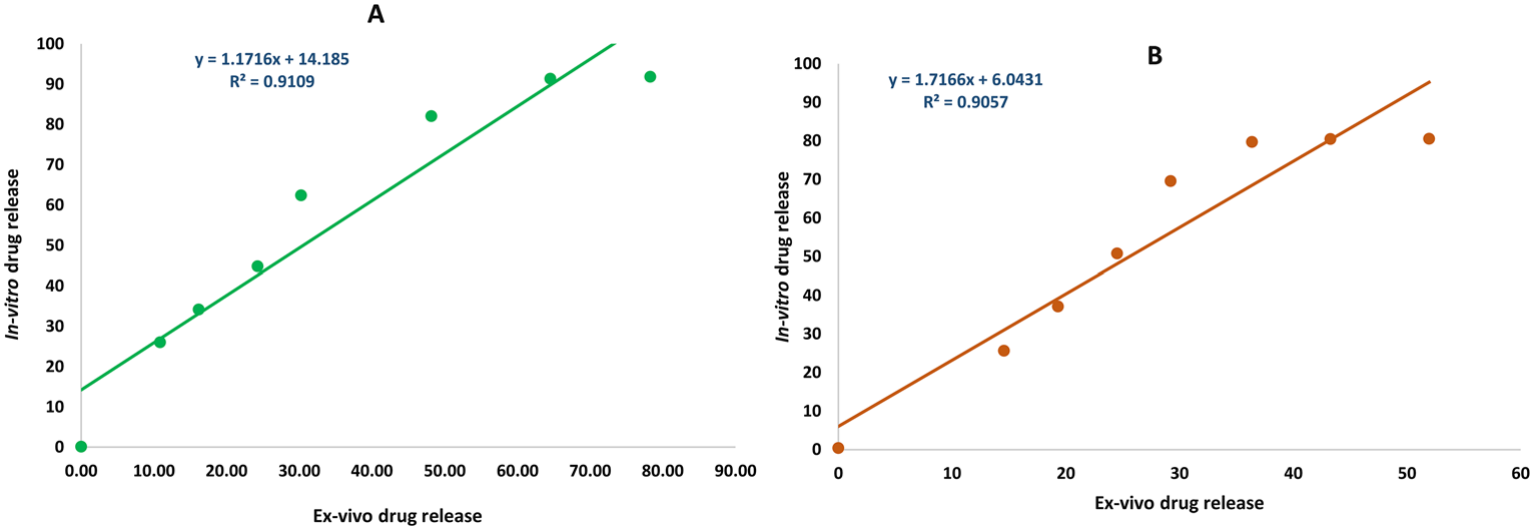
In-vitro ex vivo correlation between the cumulative drug release (**A**) Levodopa and (**B**) Carbidopa

Figure 11 shows the correlation between the in-vitro drug release profile and ex-vivo drug release for levodopa and carbidopa using the linear model [61]. The straight line and the correlation coefficient of 0.91 and 0.90 for levodopa and carbidopa demonstrate a strong correlation between the in-vitro drug release and ex-vivo drug release.

### Scanning electron microscopy (SEM) of the implant collected from release samples

**Fig. 12:**
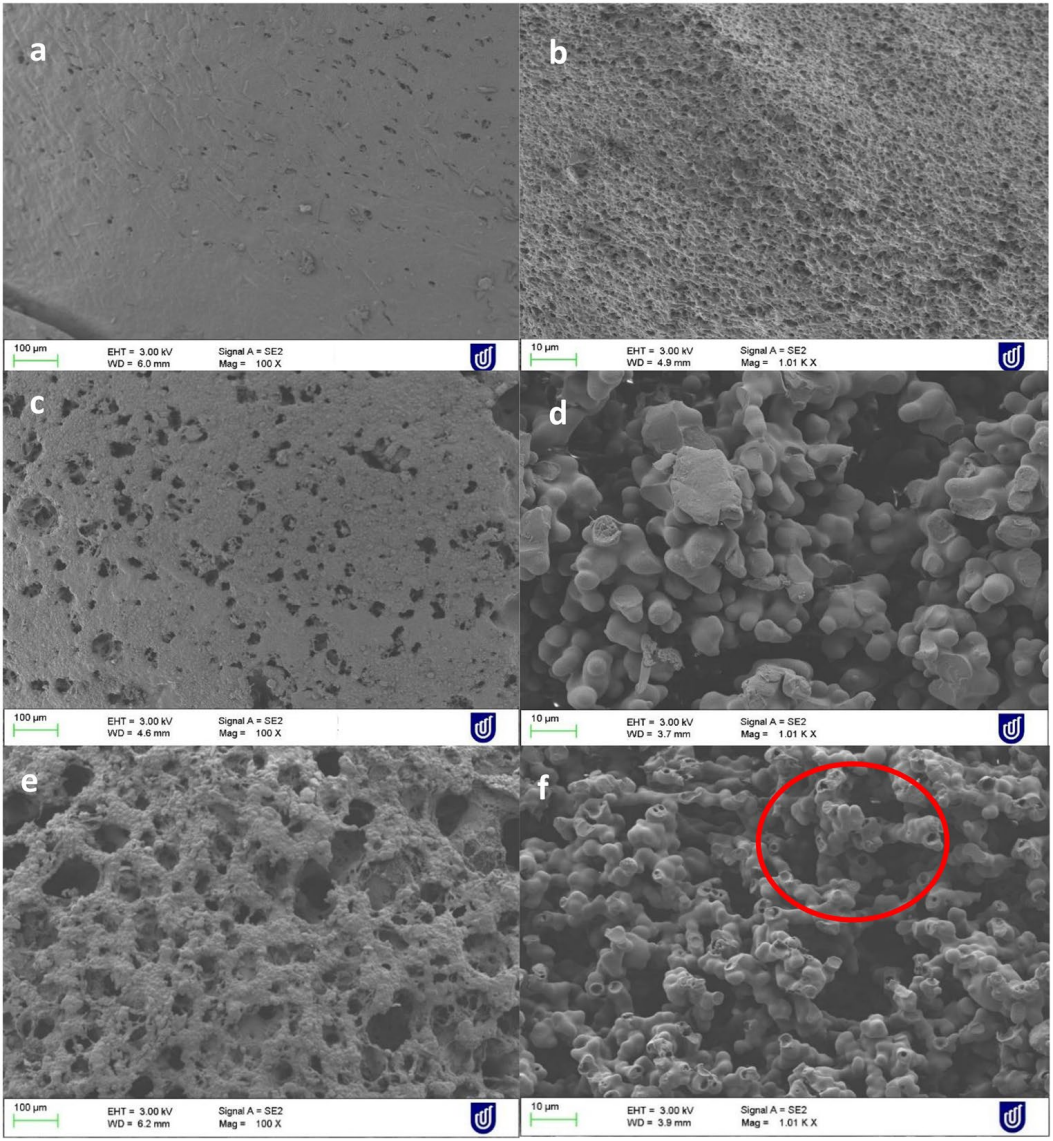
SEM images of Day 1 (**a**) surface and (**b**) cross-section, Day 3 (**c**) surface and (**d**) cross-section, and Day 7 (**e**) surface and (**f**) cross-section

SEM images of the implant samples collected on Day 1, Day 3, and Day 9 are shown in Fig. 12. Noticeable differences were observed in the surface porosity of the samples collected at different time points. As shown in Fig. 12a day 1 sample showed a compact structure on the surface as well as a cross-section. Hence the drug release in the first 8 h could be from the surface of the implant. With increasing time there has been formation of a porous structure. At day 3 pores started forming on the surface of the implant with a less compact structure on the cross section as shown in Fig. 12d. In addition, swelling of the internal structure was observed on day 3 which could be the characteristic for enhancing the drug release from the implant via diffusion towards the release media. On day 7 up to 90% drug release was observed as shown in Fig. 12e the surface became very porous with the erosion of the polymer structure highlighted in the red circle [62].

### Cell viability (MTT Assay)

**Fig. 13:**
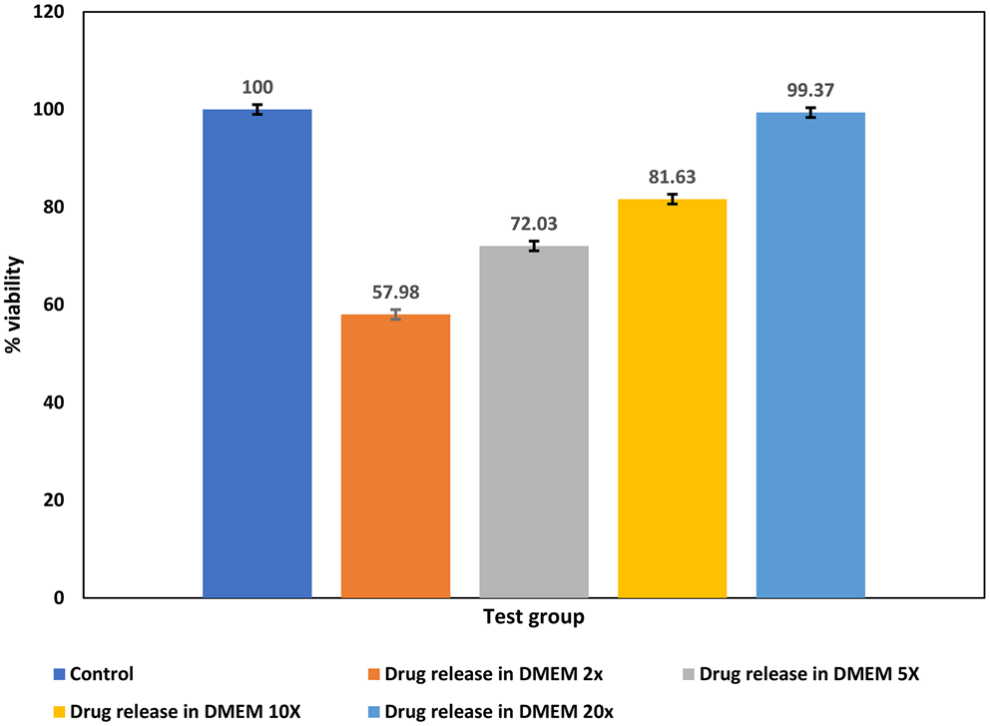
In-vitro cytotoxicity results of formulation using MTT assay on 3T3 cell lines

The amount of levodopa in the extracted sample was quantified with HPLC. The cell viability of the extracted samples was compared to that of the control group. The exposure to the levodopa extracted from the formulation in the concentration between 30 and 300 µg/ml (20x-2x dilutions) caused dose-dependent cytotoxicity in 3T3 cells as shown the Fig. 13. The cell viability was found to be decreasing with an increase in the concentration of levodopa. At 60 µg/ml (10x dilution) concentration of levodopa % cell viability was 81% which reduced to 72% for 120 µg/ml (5x dilution) concentration of levodopa. Similar findings have been reported for levodopa by other researchers showing dose-dependent cytotoxicity after exposure to 3T3 fibroblast cells for 72 h [39, 40, 63].

### In-vitro degradation studies

**Fig. 14:**
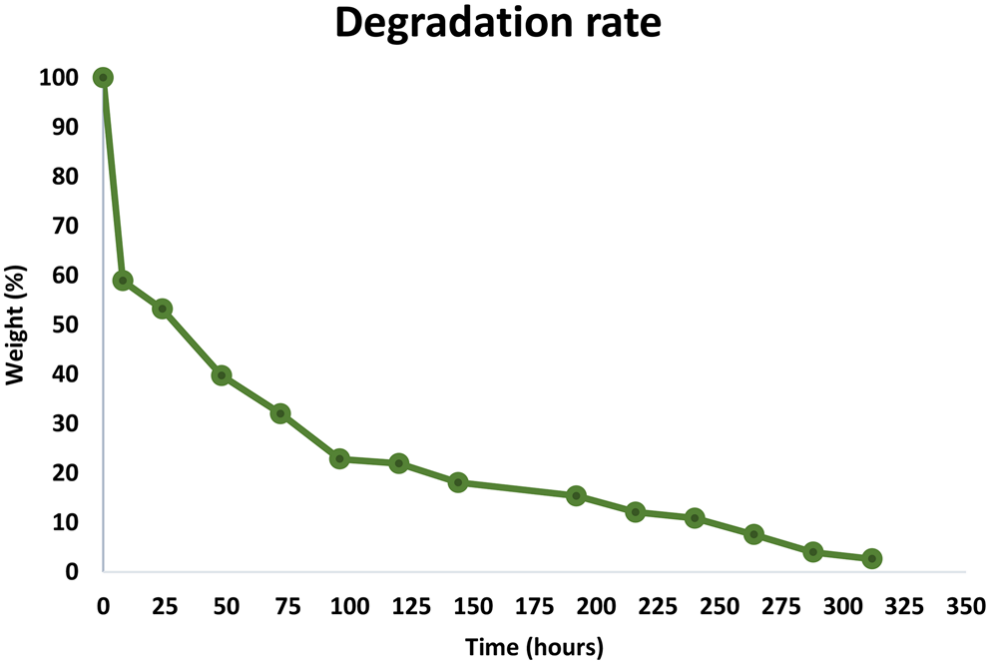
Degradation pattern for in-situ formed implant (*n* = 3)

The polymers used for preparing gels should be biodegradable, therefore it is necessary to estimate the degradation time of the polymer used. PLGA_50:50_ is biodegradable in nature but Eudragit L-100 is non-biodegradable and non-toxic, however, it solubilizes at pH 7.4. The rate of degradation will help in understanding the time required for the degradation of the polymer upon injection. The degradation studies were carried out by monitoring the weight loss of in-situ formed implant compared to that of the initial weight. As shown in Fig. 14 in the first 8 h 41.09% weight loss was observed which increased over time. After 24 h weight loss gradually increased to 46.80% followed by 60.28% on day 3. PLGA is made up of several hydroxyl acid monomers such as d-lactic acid, l-lactic acid, and glycolic acid. The hydrophobicity, release rate, and degradation rate of PLGA are determined by the lactic acid content and molecular weight, higher lactic acid content will degrade over a longer period with the slow release (46). On day 6, 78.06% weight loss was observed which indicates the biodegradable nature of the formulation. On day 7, 81.89% loss of weight was observed this loss of weight compliments the release study with 90% drug release in 7 days with SEM studies showing porous implant structure. The implants degraded completely in 13 days which was observed with a loss of 97.38% weight of the formed implant.

The degradation of PLGA occurs due to the hydrolytic cleavage of the ester linkages in the initial stage of PLGA’s degradation which results in the formation of oligomers and monomers. During this time, huge amounts of lactic and glycolic acids are formed, which causes pH values to significantly decrease. The microsphere mass reduces in the second step, and the rate of polymer chain scission could accelerate [64]. Some experts classify the two phases as erosion and degradation for drug release (48). The Krebs cycle eventually converts the generated lactic and glycolic acids to CO_2_ and water (49).

### In-vivo performance prediction

**Fig. 15:**
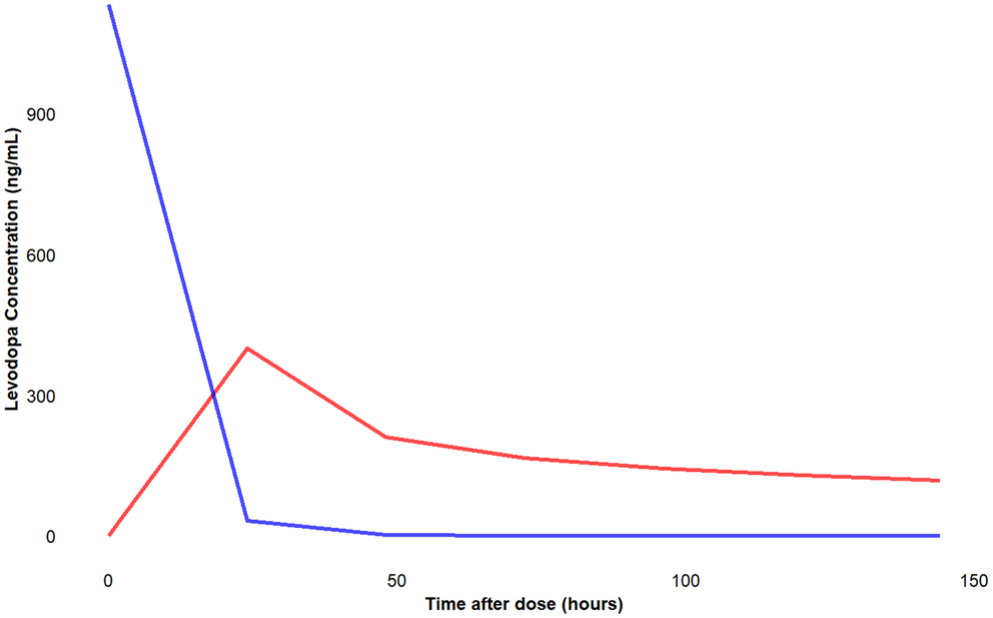
Predicted In-vivo levodopa concentration from the optimized formulation- 11.19 mg dose Red = IM formulation, Blue- IV

Convolution is one of the most commonly used approaches for predicting the plasma concentration of the drug based on in-vitro drug release data. The unit input response (UIR) was obtained from the plasma concentration-time profile of the intravenous levodopa [42]. Accordingly, the concentration of the drug in blood from the optimized in-situ forming implant was predicted for a dose of 11.19 mg levodopa. The predicted AUC _0-∞ h_ for the in-situ forming implant was 26505.5 ng/ml with C_max,_ 399.3 ng/ml, and T_max_ 24 h assuming 100% bioavailability. These predicted in-vivo concentrations provide insights into the in-vivo behavior of the developed in-situ-forming implant compared to that of intravenous formulation, which further helps in designing in-vivo studies. The optimized formulation demonstrated a sustained release profile for up to 6 days (Fig. 15). In the pharmacokinetic study reported by Yao et al., levodopa was dosed at 95 mg dose in the form of an extended-release capsule demonstrated AUC _0-∞_ 1.24 µg.h/ml and Cmax of 0.317 µg/ml with Tmax 2.8 h [65]. In comparison to the extended-release capsule, the pharmacokinetic profile for the developed in-situ forming implant demonstrated maintained plasma concentration of levodopa for up to 125 h.

## Conclusion

The in-situ implant-forming system of levodopa and carbidopa was successfully prepared by dispersing levodopa and carbidopa in the polymeric solution. The in-situ implant-forming systems are cost-effective and easy to manufacture as they do not require any specific equipment or require a tedious process for scale-up. The optimized formulation containing 26% PLGA and 6% Eudragit L-100 showed favorable in-vitro release i.e. sustained drug release up to 7 days. Up to 90% drug release was observed at day 7. A strong correlation was observed between the in-vitro drug release data and ex-vivo drug release with a correlation coefficient of 0.91 for levodopa and 0.90 for carbidopa. The surface morphology of the formed implant showed the formation of pores on the surface as well as the inner layer of the implant which compliments the release data showing diffusion of drug via pores. The formed implant demonstrated excellent in-vitro degradation with weight loss up to 80% in 7 days which was in good agreement with the in-vitro drug release profile whereas complete degradation of the implant occurs in 13 days based on the degradation studies. Further, the formulation showed low viscosity representing easy injectability, which was confirmed by syringe ability testing, formulation exhibited good syringeability via a 22 gauge needle with an injection force of 8.02 N. The predicted AUC 0-∞ h for the in-situ forming implant was 26505.5 ng/ml with Cmax, 399.3 ng/ml, and Tmax 24 h assuming 100% bioavailability. The developed formulation would be the promising drug delivery system for Parkinson’s patients which will reduce the dosing frequency in the elderly patients to once-a-week injection.

## Electronic supplementary material

Below is the link to the electronic supplementary material.

Supplementary Material 1

## Data Availability

Not applicable.
