# Supplementary material for: Development of an in-situ forming implant system for levodopa and carbidopa for the treatment of parkinson’s disease
Source: Drug Deliv Transl Res. 2025 Jun 7;15(11):4026–42. doi: 10.1007/s13346-025-01892-y (PMC12507947; doi:10.1007/s13346-025-01892-y)
Supplement: Supplementary file 1 — Supplementary Material 1 [file 13346_2025_1892_MOESM1_ESM.docx]

Convolution script (Development of an in-situ forming implant for Levodopa and Carbidopa for the treatment of Parkinsons Disease)

#library

**library**(readxl)

**library**(ggplot2)

**library**(pracma)

Time

starttime <- 0

endtime <- 144

steptime <- 24

#load data

dat_DP <- read_excel("Release.xlsx")

## New names:

## • `` -> `...4`

## • `` -> `...5`

## • `` -> `...6`

## • `` -> `...7`

## • `` -> `...8`

Plot

plot (Release~Time, data=dat_DP, type="p", col="blue", ylim=c(0,200))

#--------------------------------------------------------------------------------------------------------------

#Fit a Monoexponential curve to the release data

fitresult <- nls(Release ~ 100*(1-exp(-alpha*Time)), data = dat_DP, start = list(alpha = 0.5)) fitresult

datapred <- data.frame(Time = seq(starttime,endtime, by = steptime))

datapred$R_pred <- predict(fitresult, newdata = datapred)

#Check plotting

plot(R_pred ~ Time, data = datapred, type = "l", col = "red")

points(Release ~ Time, data = dat_DP)

#------------------------------------------------------------------------------------------------------------

## Fit a Linear model

fitresult2 <- nls(Release ~ slope*Time, data = dat_DP, start = list(slope = 1))

fitresult2

datapred <- data.frame(Time = seq(starttime,endtime, by = steptime))

datapred$R_pred <- predict(fitresult2, newdata = datapred)

datapred$CR_pred[datapred$CR_pred > 100]

## NULL

plot(R_pred ~ Time, data = datapred, type = "l", col = "red")

points(Release ~ Time, data = dat_DP)

#------------------------------------------------------------------------------------------------------------

#Compare the model fits (AIC)

TotalDose <- 11.19*1000 #dose ug

TotalDose

## [1] 11190

datapred$Rdelta <- c(0,diff(datapred$R_pred))

datapred$DosedeltaCR <- TotalDose*datapred$Rdelta/94.12792

Check the dose delta sums

DoseCheck <- sum(datapred$DosedeltaCR)

#------------------------------------------------------------------------------------------------------------

## Read IV data_Levodopa (obtained from literature)

dat.DP.IV <- read.csv("IV2.csv")

#Plot IV Data

plot (Conc~Time, data=dat.DP.IV, type="p", col="red", ylim=c(0,6000))

dat.DP.IV$CIVunit <- dat.DP.IV$Conc/50000 *#convert from ng/L to ug/L and normalise to 1ug*

#Plot normalised IV data

plot (CIVunit~Time, data=dat.DP.IV, type="p", col="red", ylim=c(0,0.2))

## Fit the iv data to get a smooth function

fitresultiv <- nls(CIVunit ~ A*exp(-alpha*Time)+B*exp(-beta*Time), data = dat.DP.IV, start = list(A = 0.1,

alpha = 0.5, B = 0.05, beta = 0.05))

fitresultiv

#Predict

dat.DP.IV$fitted <- predict(fitresultiv)

# Plot the observed data and the fitted curve

ggplot(dat.DP.IV, aes(x = Time)) +

geom_point(aes(y = CIVunit), color = "red") +

geom_line(aes(y = fitted), color = "blue") +

labs(x = "Time", y = "CIVunit", title = "Observed vs Fitted Data") +

theme_minimal()

#--------------------------------------------------------------------------------------------------------------

#Extended time points

extended_time <- data.frame(Time = seq(min(dat.DP.IV$Time), 144, by = 24))

*# Use the fitted model to predict concentrations for the extended time points*

extended_time$CIVunit_pred <- predict(fitresultiv, newdata = extended_time)

*# Ensure both data frames have the same columns*

dat.DP.IV$fitted <- predict(fitresultiv)

extended_time$CIVunit <- NA *# Adding a column for consistency*

extended_time$fitted <- extended_time$CIVunit_pred *# Rename for consistency*

*# Combine the original data with the extended predictions*

combined_data <- rbind(dat.DP.IV[, c("Time", "CIVunit", "fitted")], extended_time[, c("Time", "CIVunit", "fitted")])

*# Plot the observed data and the fitted curve including extended predictions*

ggplot() +

geom_point(data = dat.DP.IV, aes(x = Time, y = CIVunit), color = "red") +

geom_line(data = combined_data, aes(x = Time, y = fitted), color = "blue") +

labs(x = "Time", y = "CIVunit", title = "Observed vs Fitted Data with Extended Predictions") +

theme_minimal()

#------------------------------------------------------------------------------------------------------------

#IV convolution

datapred$DoseIV <- 0

datapred$DoseIV [1] <- TotalDose

input <- datapred$DoseIV

response <- extended_time$CIVunit_pred *## data obtained from IV levodopa project*

*## For some reason it requires a reversed response function*

response2 <- rev(response)

*## Convolve*

outputIV <- convolve(input, response2, type = "open")

datapred$CIV_pred <- outputIV[1:nrow(datapred)]

#------------------------------------------------------------------------------------------------------------

# #Convolution Long acting formulation

input <- datapred$DosedeltaCR

response <- extended_time$CIVunit_pred *## data obtained from IV Levodopa project*

*## For some reason it requires a reversed response function*

response2 <- rev(response)

*## Convolve*

outputCR <- convolve(input, response2, type = "open")

datapred$CCR_pred <- outputCR [1:nrow(datapred)] *#trim to just keep the time points in datapred*

*#Check the convolution by plotting*

plot(CCR_pred ~ Time, data = datapred, type = "l", col = "red")

#------------------------------------------------------------------------------------------------------------

*#Make a publication ready plot*

**library**(ggplot2)

*#In vivo concentrations*

title_text <- "Predicted in vivo Levodopa concentration for LAI containing 11.19 mg of Levodopa"

subtitle_text <- "Red = Long acting injection, Blue = IV"

*# Create the ggplot object*

plotobj <- ggplot(data = datapred) +

geom_line(aes(x = Time, y = CCR_pred), size = 1, alpha = 0.7, colour = "red")

geom_line(aes(x = Time, y = CIV_pred), size = 1, alpha = 0.7, colour = "blue") scale_y_continuous("Levodopa Concentration (ng/mL)")

scale_x_continuous("Time after dose (hours)")

labs(title = title_text, subtitle = subtitle_text) +theme_minimal(base_size = 12)

theme( plot.title = element_text(color = "black", size = 9, face = "bold", hjust = 0.5), plot.subtitle = element_text(color = "black", size = 9, hjust = 0.5, axis.title.x = element_text(color = "black", size = 9, face = "bold"),

axis.title.y = element_text(color = "black", size = 9, face = "bold"),

axis.text = element_text(color = "black", size = 9),

panel.grid.major = element_blank(),

panel.grid.minor = element_blank() )

plotobj
